# Takotsubo as Initial Manifestation of Non-Myopathic Cardiomyopathy Due to the Titin Variant c.1489G > T

**DOI:** 10.3390/medicines5030080

**Published:** 2018-07-30

**Authors:** Hans Keller, Ulrike Neuhold, Franz Weidinger, Edmund Gatterer, Claudia Stöllberger, Klaus Huber, Josef Finsterer

**Affiliations:** 1 2nd Medical Department with Cardiology and Intensive Care Medicine, Krankenanstalt Rudolfstiftung, 1030 Wien, Austria; hans.keller@wienkav.at (H.K.); franz.weidinger@wienkav.at (F.W.); Edmund.gatterer@wienkav.at (E.G.); Claudia.stoellberger@wienkav.at (C.S.); 2 Medical Department, Krems University Hospital, Krankenhaus Krems an der Donau, 3500 Krems an der Donau, Austria; Ulrike.neuhold@lknoe.at; 3 Genetic Laboratory, SMZO, 1220 Vienna, Austria; klaus.huber@wienkav.at; 4 Krankenanstalt Rudolfstiftung, Postfach 20, 1180 Vienna, Austria

**Keywords:** dilated cardiomyopathy, ventricular arrhythmias, heart failure, myopathy, titin, muscular dystrophy

## Abstract

**Background**: Whether patients with subclinical cardiomyopathy (CMP) are more prone to experience Takotsubo syndrome (TTS) than patients without CMP, is unknown. We present a patient with TTS as the initial manifestation of a hitherto unrecognized genetic CMP. **Method**: case report. **Results**: At age 55 after the unexpected death of her father, a now 61-year-old female had developed precordial pressure. Work-up revealed moderately reduced systolic function, dyskinesia of the interventricular septum, and indications for a TTS. Coronary angiography was normal but ventriculography showed TTS. Cardiac MRI confirmed reduced systolic function and TTS. TTS resolved without treatment and sequelae. At age 57 atrial fibrillation was recorded. After deterioration of systolic function at age 59 dilated CMP was diagnosed. Despite application of levosimendan, sacubitril, valsartan, and ivabradine, complete remission could not be achieved. Upon genetic work-up by means of a gene panel, the heterozygous mutation c.1489G > T (p. E497X) in exon 9 of the *titin* gene was detected and made responsible for the phenotype. Neurological work-up precluded involvement of the skeletal muscles. The further course was complicated by ventricular arrhythmias, requiring implantation of an implantable cardioverter defibrillator (ICD). Conclusions: previously subclinical CMP may initially manifest as TTS. Since patients with titin CMP are at risk of developing ventricular arrhythmias and thus to experience sudden cardiac death, appropriate anti-arrhythmic therapy needs to be established.

## 1. Novel Insights

Previously, subclinical dCMP due to a *titin* variant may initially manifest as Takotsubo syndrome.

Patients with titin cardiomyopathy are prone to develop ventricular arrhythmias.

## 2. Established Facts

Titin variants may manifest in the heart as hypertrophic cardiomyopathy.

Titin mutations may exclusively manifest in the heart without skeletal muscle involvement.

## 3. Introduction

Takotsubo syndrome (TTS), also known as stress cardiomyopathy (CMP) or broken-heart syndrome, is characterized by stress-triggered acute systolic dysfunction, clinically, electrocardiographically, and chemically mimicking myocardial infarction [1]. Whether patients with subclinical, genetic CMP are more prone to experience TTS than patients without CMP, is unknown. No reports about TTS as initial manifestation of a primary CMP are available. Here we report with written consent a patient with TTS as the initial clinical manifestation of a hitherto unrecognized genetic CMP.

## 4. Case Report

**Figure 1:**
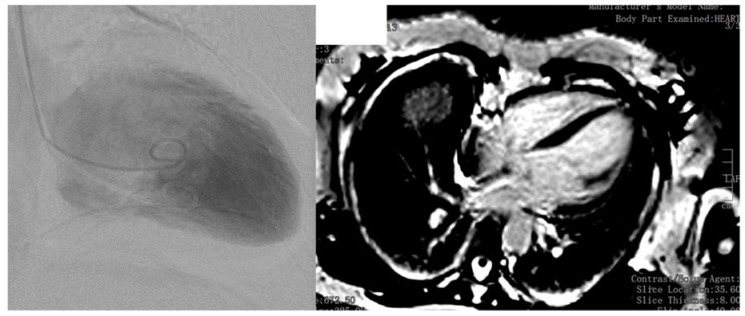
Ventriculography showing ballooning of the apical region of the left ventricle (**left panel**). Cardiac magnetic resonance imaging, 2 years after the initial event, showing no signs of late gadolinium enhancement (**right panel**).

The patient is a 61-year-old Caucasian female, height 168 cm, weight 58 kg, with an uneventful previous history until age 55, when she developed precordial pressure after exposure to psychosocial stress after the unexpected death of her father. ECG showed left anterior hemiblock, missing R-progression until V4, and flat T-waves in III, aVL, and V1. Echocardiography revealed moderately reduced systolic function, dyskinesia of the interventricular septum, and regional wall motion abnormalities, indicative of TTS. Coronary angiography was normal but ventriculography was indicative of TTS (Figure 1). Cardiac MRI (cMRI) revealed a reduced systolic function with a left ventricular ejection fraction (EF) of 40%. Stress testing revealed reduced physical capacity. TTS resolved after a few days without therapy. The family history was positive for sudden death of her brother at age 66 and her grandmother from the mother’s side at age 77. Her mother, aged 85 suffered from heart failure.

At age 57 tachycardious atrial fibrillation (AF) and isolated ventricular ectopic beats were recorded, which resolved spontaneously. ProBNP was 866 ng/L (*n*, 0–247 ng/L). The EF on cMRI had slightly improved (48%) compared to the previous cMRI. After initiation of a neurohumoral therapy with carvedilol, angiotensin-converting enzyme inhibitors (ACEI), and a statin, systolic function improved, stress test became normal, and proBNP declined to 152 ng/L. At age 59 the EF deteriorated again to 48% and the LVEDD to 59 mm. Despite re-establishing β-blockers, the EF further decreased to 40%, the LVEDD increased to 68 mm, and the proBNP to 1058 ng/L. ECG showed stable sinus rhythm but there was easy fatigability upon psychosocial stress.

After pneumonia at age 60, severe heart failure developed with an EF of 18%. Echocardiography showed mitral insufficiency and pulmonary hypertension. Coronary angiography was normal again. Myocarditis was excluded upon cMRI (Figure 1). Levosimendan was given once, followed by sacubitril and valsartan in combination and ivabradine. The latter had to be discontinued after two months because of a suspected arrhythmogenic effect. Since the patient initially refused implantation of an implantable cardioverter defibrillator (ICD), a LifeVest^®^ was prescribed. Already one day after dismissal, the LifeVest^®^ delivered an appropriate shock because of ventricular fibrillation. After admission, three further episodes of ventricular fibrillation occurred, which were all terminated by adequate LifeVest^®^ shocks. Because of a suspected pro-arrhythmogenic effect, procoralan was discontinued and a therapy with amiodarone begun. Additionally, an ICD was implanted, genetic investigations initiated, and the patient was scheduled for heart transplantation (HTX).

Genetic testing by means of a gene panel covering 40 genes associated with dilated CMP (dCMP) revealed the heterozygous mutation c.1489G > T (p. E497X) in exon 9 of the *titin* gene. The neurological history was noteworthy for intense myalgias during gripal infections since years and sore muscles during one month with pneumonia. Since clinical neurologic exam and creatine-kinase (CK) were normal, no further invasive work-up for myopathy was conducted. At discharge she was on a therapy with sacubitril (97 mg/d), valsartan (103 mg/d), nebivolol (1,25 mg/d), amiodarone (50 mg/d), spironolactone (25 mg/d), furosemide (40 mg/d), and duloxetine (30 mg/d).

## 5. Discussion

The presented case is interesting for dCMP due to the *titin* mutation c.1489C > T, for TTS as the initial manifestation of titin CMP, and for the absence of skeletal muscle involvement.

Heart failure is a major health burden and affects approximately 40 million people worldwide [2]. One of the major causes of heart failure is dCMP [2], which is defined by ventricular chamber enlargement and systolic dysfunction without left ventricular wall thickness, leading to progressive heart failure [3]. dCMP occurs with a prevalence of 1:2500 and is responsible for half of the cases with heart failure [4]. About one third of the dCMPs are due to genetic causes [2]. Mutations in >60 genes have been identified so far, which are made responsible for dCMP [3].

One of the dCMP genes is the *titin* gene, located on chromosome 2q31. The *titin* gene encodes titin, the largest known protein in biology, spanning half the cardiac and muscle sarcomere and, as such a basic structural and functional unit of striated muscles [3]. Titin is built up of 38,000 aminoacids from 364 exons [5]. Mutations in the *titin* gene can cause dCMP, hypertrophic CMP, myopathy, or overlap forms [6]. There are indications that a single *titin* mutation may present phenotypically with different manifestations in the same family or different families [7]. Mutations in the *titin* gene are detectable in 20–25% of the hereditary forms of dCMP and in 18% of the patients with the sporadic type of dCMP [8,9,10]. How many of the *titin* mutations manifest with dCMP, hCMP, myopathy or overlap phenotypes is unknown. dCMP due to the *titin* mutation c.1489G > T has not been reported so far. Nonetheless, the new variant was regarded as pathogenic since pathogenic mutations in the near surrounding of this variant had been reported. However, in case of additional stress, systolic dysfunction or heart failure may ensue. There are also indications that truncating titin variants represent a modifier of hypertrophic CMP and an increased risk of cardiovascular death [11]. Subclinical truncating *titin* variants may be also associated with chemotherapy-induced CMP [12]. Important to know is that CMP may be associated with noncompaction [13]. In animal models of titin CMP, it has been shown that the genetic defect can be compensated by compensatory enhanced energy production.

Concerning titin-related myopathy, *titin* mutations most frequently manifest in the skeletal muscle as limb-girdle muscular dystrophy type 2J [14], recessive congenital myopathies, or late-onset dominant distal myopathy [7]. In some of these patients cardiac involvement may occur, whereas other patients do not present with cardiac compromise [7]. Cardiac involvement in titin myopathy includes arrhythmias and CMP [15]. In a study of 22 patients carrying a *titin* variant, 32% had arrhythmias (sustained atrioventricular tachycardia, AF, sinus bradycardia, supraventricular ectopic beats) and 18% had CMP [15]. Titin myopathy may also go along with features of myositis on muscle biopsy [15].

In conclusion, this case shows that a previously subclinical dCMP due to a *titin* variant may initially manifest as TTS. Patients with titin CMP seem to be at risk of developing ventricular arrhythmias and thus to experience sudden cardiac death, why appropriate anti-arrhythmic therapy needs to be established. The LifeVest^®^ is the most appropriate tool to effectively bridge the interval between indication and implantation of an ICD but in-hospital telemetry or anti-arrhythmic drugs could be an alternative.
